# Expression of biomarker genes of differentiation in D3 mouse embryonic stem cells after exposure to different embryotoxicant and non-embryotoxicant model chemicals

**DOI:** 10.1016/j.dib.2015.09.015

**Published:** 2015-09-30

**Authors:** Andrea C. Romero, Eva del Río, Eugenio Vilanova, Miguel A. Sogorb

**Affiliations:** Unidad de Toxicología y Seguridad Química, Instituto de Bioingeniería, Universidad Miguel Hernández de Elche, Avenida de la Universidad s/n, 03202 Elche, Spain

## Abstract

There is a necessity to develop *in vitro* methods for testing embryotoxicity (Romero et al., 2015) [1]. We studied the progress of D3 mouse embryonic stem cells differentiation exposed to model embryotoxicants and non-embryotoxicants chemicals through the expression of biomarker genes. We studied a set of 16 different genes biomarkers of general cellular processes (*Cdk1, Myc, Jun, Mixl, Cer* and *Wnt3*), ectoderm formation (*Nrcam, Nes, Shh* and *Pnpla6*), mesoderm formation (*Mesp1, Vegfa, Myo1e* and *Hdac7*) and endoderm formation (*Flk1* and *Afp*). We offer dose response in order to derive the concentration causing either 50% or 200% of expression of the biomarker gene. These records revealed to be a valuable end-point to predict *in vitro* the embryotoxicity of chemicals (Romero et al., 2015) [1].

Specifications Table

**Table:** 

Subject area	Toxicology, embryotoxicity, developmental toxicity
More specific subject area	Alternative testing methods, embryonic stem cell, cell differentiation
Type of data	2 Tables, 1 Scheme and 7 figures
How data was acquired	Quantitative real time PCR (StepOnePlus Real-Time PCR System (Applied Biosystems) equipment)
Data format	Analyzed and plotted data
Experimental factors	D3 mouse embryonic stem cells under spontaneous differentiation were exposed during 5 days to several concentrations of the model embryotoxic chemicals.
Experimental features	After exposure cells were lysed, mRNA was extracted and the expression of the biomarker genes analyzed
Data source location	Elche, Alicante (Spain)
Data accessibility	Data is provided in the article

**Value of the data**

• We offer to the readers examples of widely used chemicals with different *in vivo* embryotoxicity potency.
• We offer to the readers the primer sequence and their respective annealing temperatures to assay using Power SYBR Green methodology the quantitative real time PCR the expression of 6 different genes (5 biomarkers of differentiation plus a house-keeping).
• We show as the treatments do not affect the expression of the house-keeping gene, which is an unavoidable requirement for validating the quantification of gene expression.
• We show doses–responses of model chemicals that allow deriving the concentrations causing either 50% or 200% of expression of the biomarker genes.

## 1 Data

We needed to select model chemicals with different embryotoxicity in order to develop a cellular method for testing embryotoxicity based on the alterations of the differentiation of D3 mouse embryonic stem cells. We finally selected our model chemicals (Table 1) among those that were previously used in the pre-validation or validation study of an embryonic stem cell method sponsored by the European Union Reference Laboratory for Alternatives to Animal Testing and by other papers dealing with the development of *in vitro* methods for testing embryotoxicity [2–4].

We needed to assay the effect of the selected chemicals (Table 1) on the alterations of D3 cells monitoring changes in biomarker genes. For that, we used quantitative PCR with Power SYBR Green methodology for 5 biomarker genes (plus in house-keeping gene). We designed for this purpose the primers shown in Table 2. Table 2 is also displaying annealing temperatures of such primers.

**Scheme 1:**
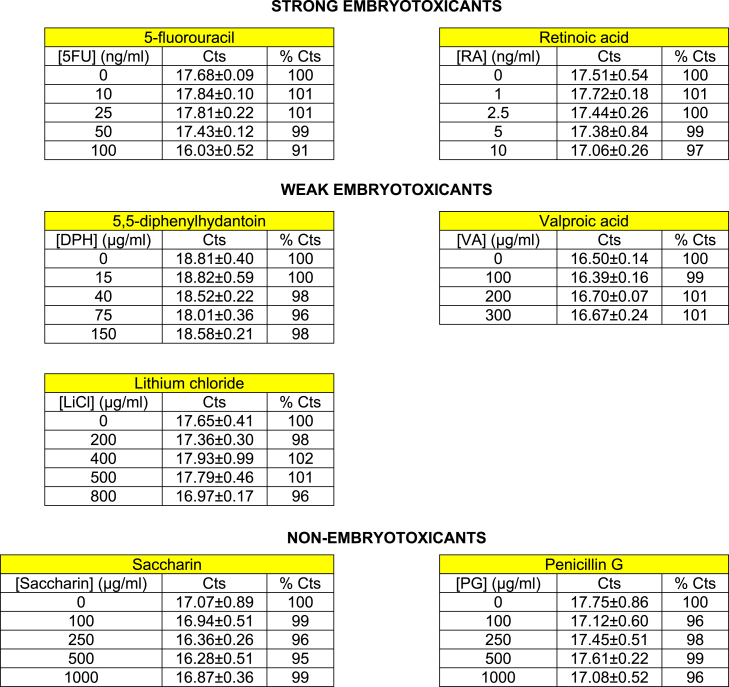
CTs recorded during actin expression. It is displayed number of number of thermal cycles needed to reach the threshold of fluorescence previously set during quantitative real time PCR experiments. It is displayed for each treatment the mean±s.d. for three biological replicates.

In order to check if the chemicals alter the expression of the house-keeping gene (β-actin) we determined that there were no statistical significant differences among the number of thermal cycles of control samples and samples exposed to all the tested concentrations of all model chemicals listed in Table 1 (Scheme 1). These findings are needed in order to validate further results with the biomarker genes.

**Fig. 1:**
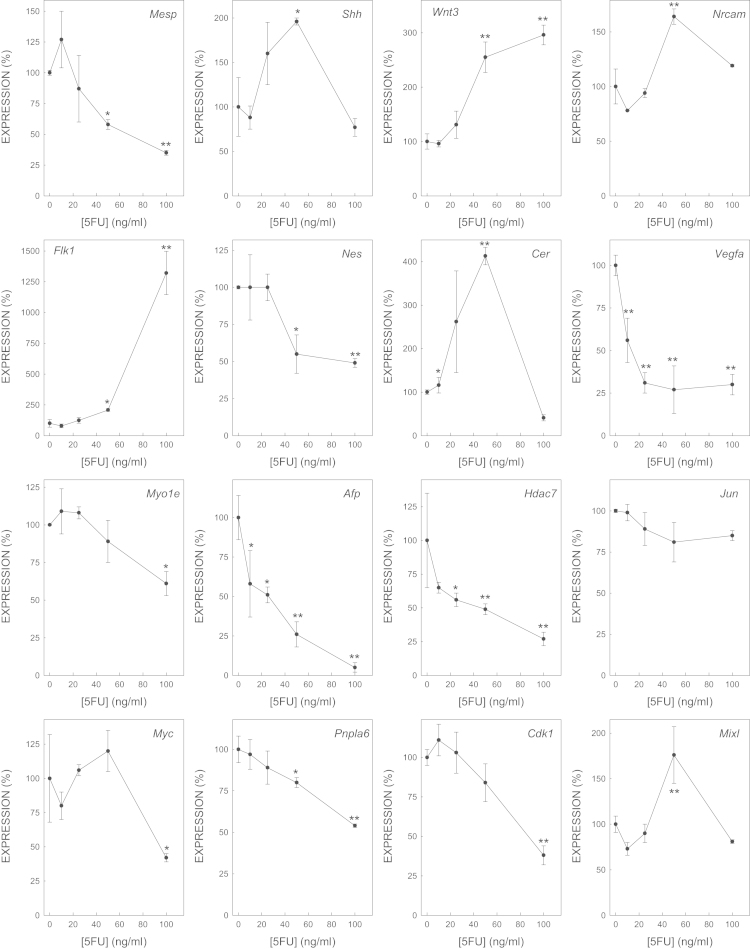
Effect of 5FU on the expression of biomarker genes.

**Fig. 2:**
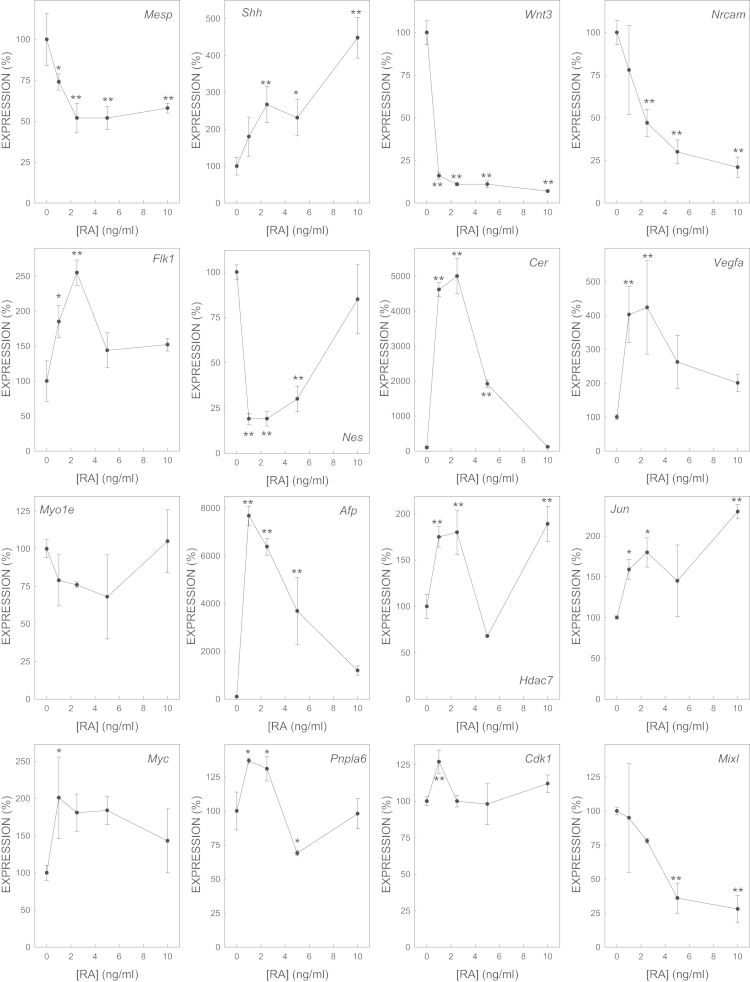
Effect of RA on the expression of biomarker genes.

**Fig. 3:**
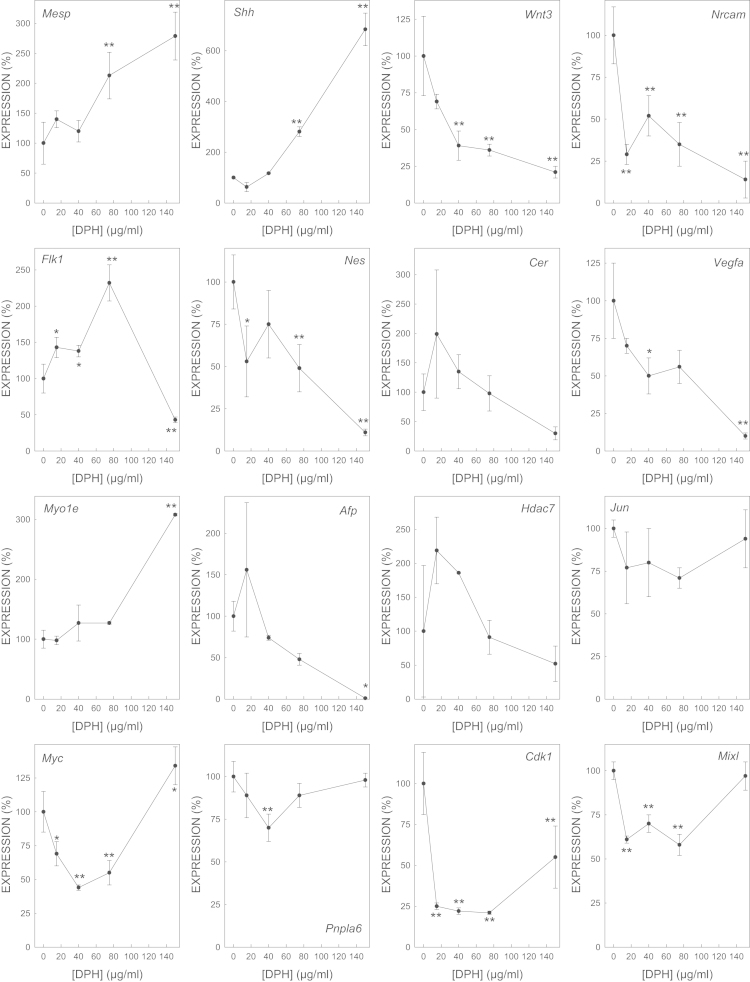
Effect of DPH on the expression of biomarker genes.

**Fig. 4:**
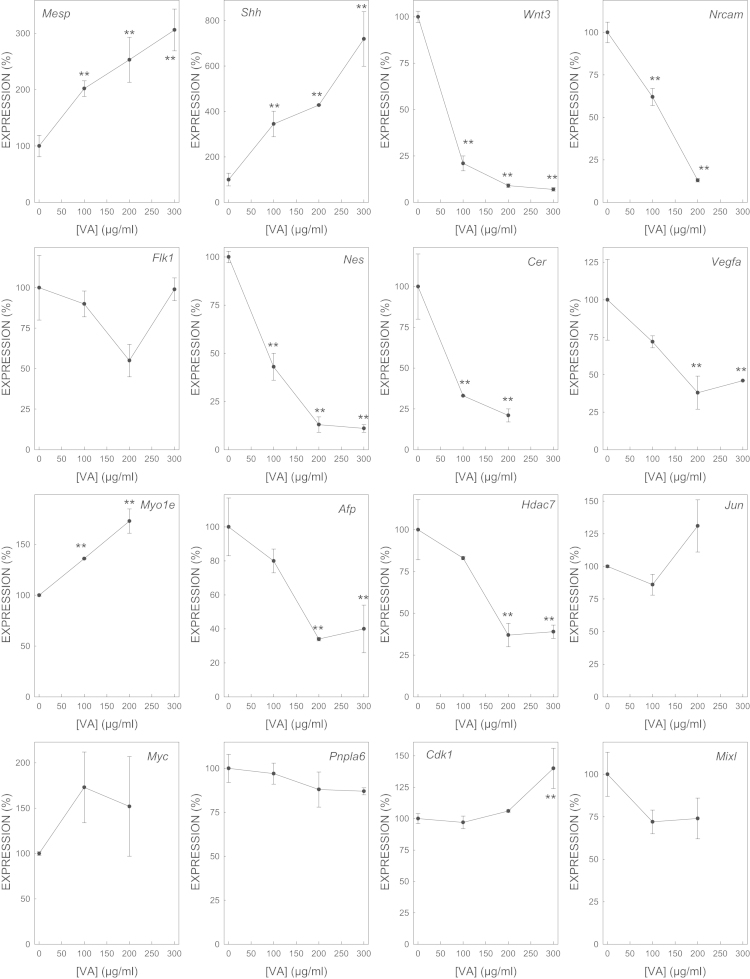
Effect of VA on the expression of biomarker genes.

**Fig. 5:**
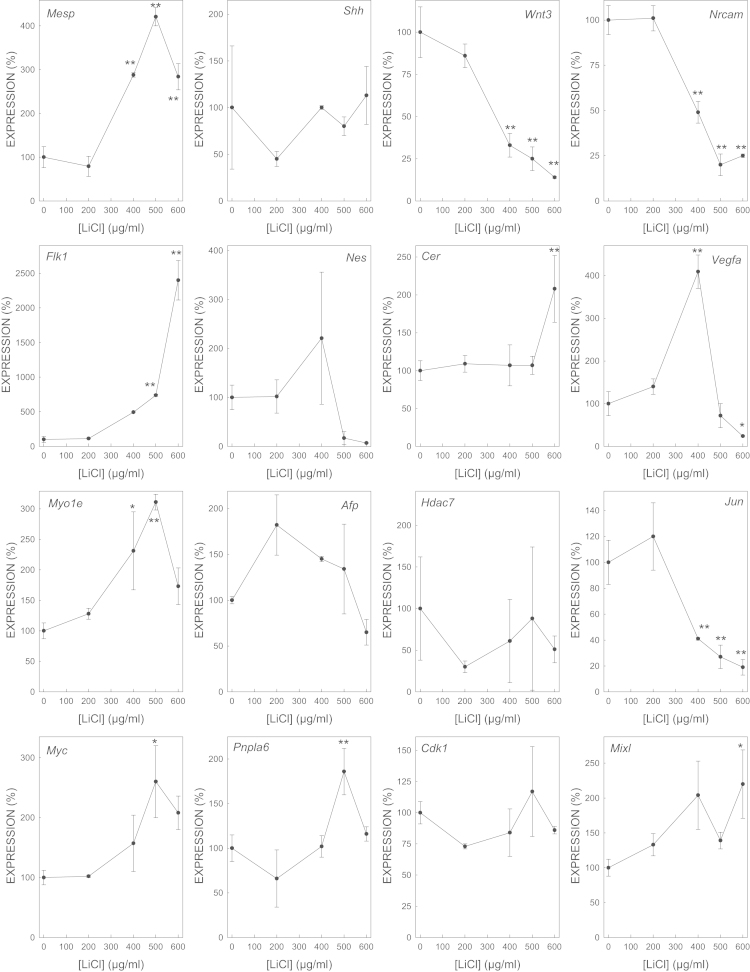
Effect of LiCl on the expression of biomarker genes.

**Fig. 6:**
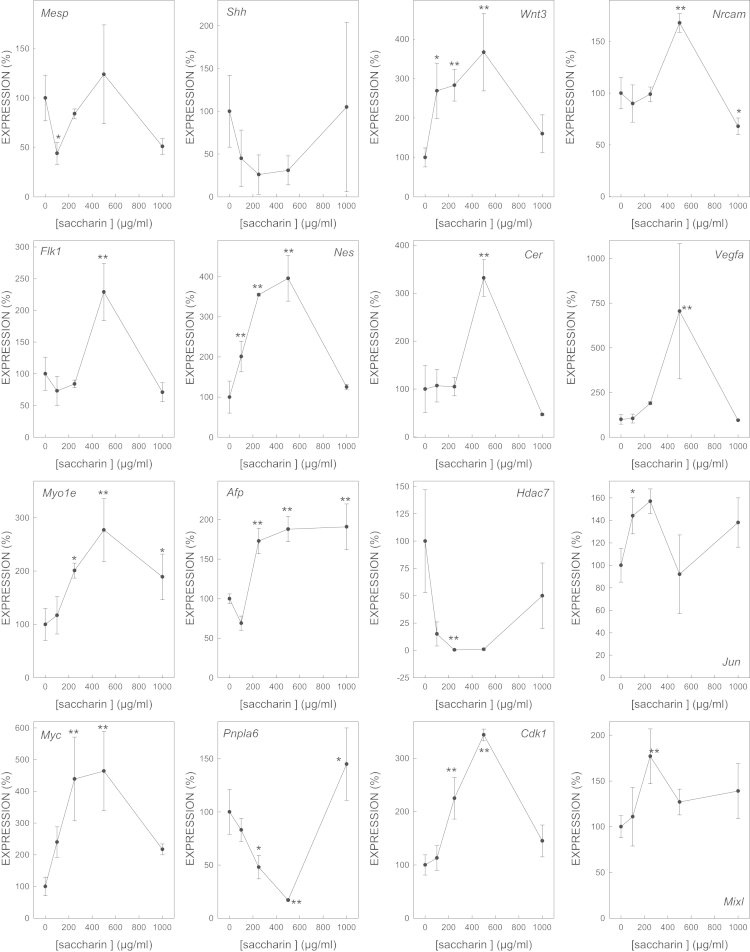
Effect of saccharin on the expression of biomarker genes.

**Fig. 7:**
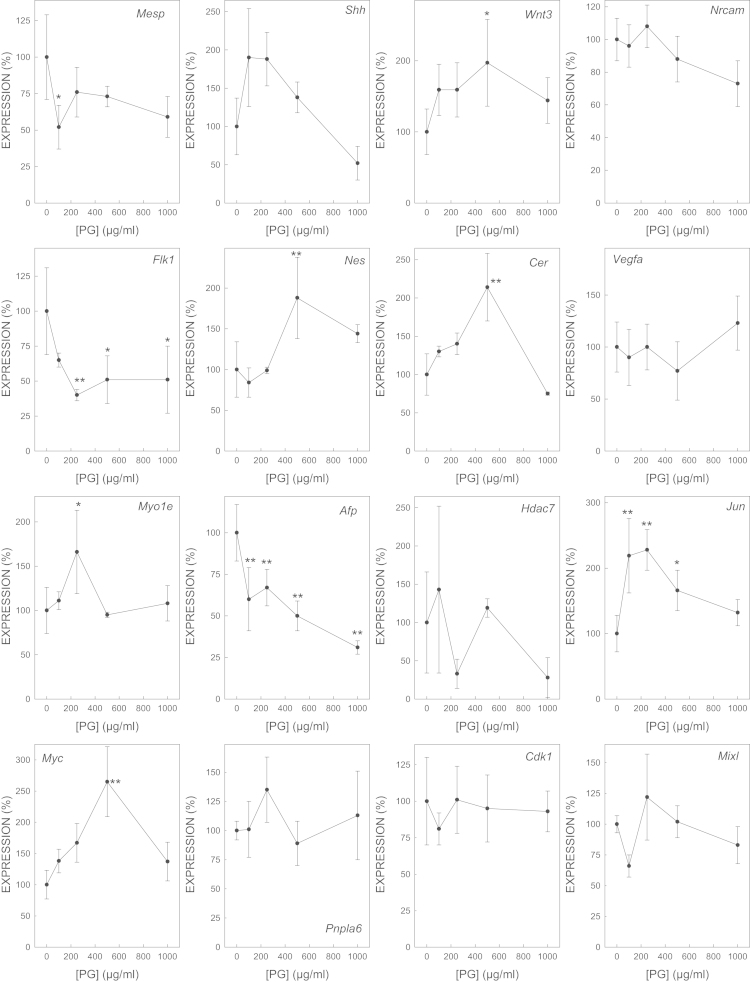
Effect of PG on the expression of biomarker genes.

We determined the effect of gene expression of biomarker genes of all the selected model embryotoxicants (Figs. 1–7). The dose–response plots were used to derive ECD50 or ECD200, which were used as end-points for enhancing the performance of embryonic stem cell methods for testing embryotoxicity [1].

## 2 Experimental design, materials and methods

D3 cells cultured on monolayer under spontaneous differentiation were exposed to several concentrations of the strong embryotoxicants 5-fluorouracil (Fig. 1) and retinoic acid (Fig. 2); of the weak embryotoxicants 5,5-diphenylhydantoin (Fig. 3), valproic acid (Fig. 4) and LiCl (Fig. 5); and of the non-embryotoxicants saccharin (Fig. 6) and penicillin G (Fig. 7) for 5 days. At the end of exposure, cells were lysed, RNA was extracted and retrotranscribed to cDNA, and each gene was amplified and quantified by quantitative real time PCR as previously described [1,5,6] and using to 2^−ΔΔCt^ calculations [7] and β-actin as a house-keeping control gene The expression of each gene was normalized against the expression of this same gene in the control (non-exposed) cells. The mean±s.d. of three independent biological replicates run in the experiment is shown. (*=statistically different form control for at least *p*<0.05 in Dunnett test; **=statistically different form control for at least *p*<0.01 in Dunnett test.)

## Figures and Tables

**Table 1:** Embryotoxic model chemicals.

Chemical	CAS number	Supplier	Catalog reference	Purity (%)	*in vivo* embryotoxicity
5-fluorouracil	51-21-8	Sigma	F6627	>99	Strong
Retinoic acid	302-79-4	Sigma	R2625	>98	Strong
LiCl	7447-41-8	Sigma	L9650	>99	Weak
5,5-diphenylhydantoin	630-93-3	Sigma	D4505	>99	Weak
Valproic acid	99-66-1	Fluka	05194	>98	Weak
Saccharin	82385-42-0	Sigma	S6047	>99	Non
Penicillin G	69-57-8	Fluka	13752	>98	Non

**Table 2:** Primer sequences and annealing temperatures used in the quantitative real time PCR experiments with Power SYBR Green methodology.

Gene	5′ – 3′ oligo	3′ – 5′ oligo	*T* (°C)
Nes	GCTTTCCTGACCCCAAGCTG	GGCAAGGGGGAAGAGAAGGA	61
Flk1	CAGCCAGACAGACAGTGGGATGGTC	CCGAGGCCACAGACTCCCTGCTT	61
Afp	GCTGCAAAGCTGACAACAAG	GGTTGTTGCCTGGAGGTTTC	63
Hdac7	CCATGTTTCTGCCAAATGTTTTGG	GCCGTGAGGTCATGTCCACC	63
Vegfa	CGTTCACTGTGAGCCTTGTTCAG	GCCTTGCAACGCGAGTCTGT	60
Actin	CCCTAGGCACCAGGGTGTGA	TCCCAGTTGGTAACAATGCCA	62
